# Stakeholders’ experiences of comprehensive geriatric assessment in an inpatient hospital setting: a qualitative systematic review and meta-ethnography

**DOI:** 10.1186/s12877-023-04505-w

**Published:** 2023-12-08

**Authors:** Íde O’Shaughnessy, Christine Fitzgerald, Christina Hayes, Aoife Leahy, Margaret O’Connor, Damien Ryan, Denys Shchetkovsky, Fiona Steed, Leonora Carey, Colin Quinn, Elaine Shanahan, Rose Galvin, Katie Robinson

**Affiliations:** 1 School of Allied Health, Faculty of Education and Health Sciences, Ageing Research Centre, Health Research Institute, University of Limerick, Limerick, Ireland; 2 Department of Ageing and Therapeutics, University Hospital Limerick, Dooradoyle, Limerick, Ireland; 3 School of Medicine, Faculty of Education and Health Sciences, University of Limerick, Limerick, Ireland; 4 Emergency Department, Limerick EM Education Research Training (ALERT), University Hospital Limerick, Dooradoyle, Limerick, Ireland; 5 Department of Health, Baggot Street, Dublin, Ireland; 6 Department of Occupational Therapy, University Hospital Limerick, Dooradoyle, Limerick, Ireland

**Keywords:** Older adults, Comprehensive geriatric assessment, Inpatient, Qualitative evidence synthesis, Meta-ethnography

## Abstract

**Background:**

Comprehensive geriatric assessment (CGA) is considered the gold standard approach to improving a range of outcomes for older adults living with frailty admitted to hospital. To date, research has predominantly focused on quantitative syntheses of the international evidence with limited focus on qualitative synthesis of stakeholder perspectives. This review aims to resolve this research gap by identifying and synthesising qualitative studies reporting multiple stakeholders’ experiences of inpatient CGA.

**Methods:**

A systematic search of five electronic databases was conducted. Qualitative or mixed methods studies that included qualitative findings on the experiences of CGA in an inpatient hospital setting from the perspective of healthcare professionals (HCP), older adults, and those important to them were included. The protocol was registered on PROSPERO (Registration: CRD42021283167) and the 10-item Critical Appraisal Skills Programme checklist was used to appraise the methodological quality of included studies. Results were synthesised as a meta-ethnography.

**Results:**

Eleven studies, which reported on the experiences of 153 HCPs, 91 older adults and 57 caregivers were included. The studies dated from 2011 to 2021 and three key themes were identified: (1) HCPs, older adults and caregivers report conflicting views on CGA as a holistic process, (2) most HCPs, but only some older adults and caregivers view CGA goalsetting and care planning as collaborative, and (3) all stakeholders value care continuity during the transition from hospital to home but often fail to achieve it.

**Conclusion:**

While HCPs, older adults, and caregivers’ values and ambitions related to CGA broadly align, their experiences often differ. The identified themes highlight organisational and relational factors, which positively and negatively influence CGA practices and processes in an inpatient hospital setting.

**Supplementary Information:**

The online version contains supplementary material available at 10.1186/s12877-023-04505-w.

## Background

Population ageing has transformed the demand for health care worldwide. Older adults accessing acute hospital care often present with multiple comorbidities, cognitive impairment, polypharmacy, and functional decline [1, 2]. This accumulation of deficits is an indication for assessment of frailty [3], which is conceptualised as a state of increased vulnerability resulting from age-associated decline in physiological reserves [4] and is predictive of multiple adverse outcomes during hospitalisation [5–7]. Comprehensive geriatric assessment (CGA) is considered the gold standard approach for hospital-based health care for older adults living with frailty [8]. CGA is a multi-dimensional therapeutic and diagnostic process that is focused on improving the quality of life and health outcomes of older adults living with frailty [9, 10].

To date, research conducted on the impact of CGA for older adults admitted to hospital has predominantly focused on quantitative syntheses of the international evidence. Compared to ‘usual care’, CGA has been shown to have positive effects on key clinical and process outcomes [8, 11, 12]. The CGA process in hospital settings increases the likelihood of being alive and living at home at three to 12 months’ follow-up [8, 11], decreases the likelihood of admission to a nursing home at three to 12 months’ follow-up [8, 12], and is associated with a reduction in functional decline at hospital discharge [12] and at six-month follow-up [11]. This primary focus on quantitative outcome measurement is further illuminated in findings from an umbrella review, which aimed to describe the key elements, principal outcome measures, and characteristics of the main beneficiaries of inpatient CGA [13]. Of the 13 systematic reviews included, only two included patient reported outcome or experience measures such as quality of life or active listening. Given that CGA is underpinned by a person-centred approach to care [14], the authors recommended greater inclusion of patientreported outcome and/or experience measures, which may guide healthcare professionals (HCP) in re-focusing assessment and intervention processes around older adults’ priorities and preferences.

Optimisation of CGA delivery across different patient populations with multiple comorbidities was identified as one of the top 10 research priorities by older adults and those important to them in a recent James Lind Alliance Priority Setting Partnership [15]. Qualitative research has identified important contradictions between the aims of CGA and patients’ experiences, for example, a study, which explored older adults’ experiences of CGA in an outpatient setting, revealed a dominant focus on illness symptoms and limitations related to ageing [16]. It is increasingly important to understand older adults’ perspectives as international healthcare systems continue to evolve and reconfigure to meet increased demand for resources [17–19].

A number of qualitative studies exploring the experience of CGA in an inpatient hospital setting from the perspective of multiple stakeholders have been published; however, to our knowledge, no qualitative evidence synthesis has been completed on the topic. Our study aims to: (1) systematically search the qualitative literature to identify studies exploring HCPs, older adults and those important to them experiences of CGA in an inpatient hospital setting, and (2) perform a meta-ethnography to synthesise the included studies with a view to developing new insights reflective of all stakeholders’ experiences.

## Methods

### Design

A meta-ethnographic synthesis, informed by Noblit and Hare’s [20] seven-phase process of the qualitative evidence was selected to move beyond aggregation and description of the evidence to engagement in interpretative analysis and generation of new insights [21]. The eMERGe guidelines [22] were adhered to in the conduct and reporting of this meta-ethnography with a completed checklist presented in Additional file 1. The original protocol for the synthesis was registered on PROSPERO (CRD42021283167).

### Search strategy

A systematic search of five electronic databases (CINAHL, PsychArticles, PsychInfo, MEDLINE, Social Sciences) was completed using “geriatric assessment” and “qualitative research” as keywords alongside exploded subject headings in May 2023; no limit was placed on date of publication. These databases were selected for inclusion of qualitative studies and health research. We only included peer-reviewed publications i.e., grey literature and abstracts were excluded, and studies were limited to those published in English. The search string can be found in Additional file 2. References generated from the search strategy were exported into Endnote X9 software and duplicates deleted. Reference lists of included studies were searched for additional papers.

### Eligibility criteria

We included studies that used recognised qualitative methods of data collection and analysis, which reported the experiences and/or perceptions of stakeholders involved in CGA within an inpatient hospital setting. Stakeholders included HCPs, older adults, and those important to them e.g., family members and/or caregivers. The definition of CGA described by Parker et al. [13] as a ‘*multidimensional, multidisciplinary process, which identifies medical, social and functional needs, and the development of an integrated/co-ordinated care plan to meet those needs*’ was used as the reference standard when determining the eligibility of studies for inclusion. Studies that used mixed methods where qualitative data could be extracted were also included. Studies that described CGA within an inpatient surgical, psychiatry, or long-term residential setting were excluded due to the expected differences in recovery trajectory and thus experience of hospitalisation.

### Screening

Two reviewers (ÍO’S and KR) independently conducted title and abstract screening against inclusion criteria. Disagreements were resolved through discussion and consensus with a third reviewer (CF), where necessary. Full text articles were independently screened by two reviewers (ÍO’S and KR) for final decisions regarding inclusion, with disagreement resolved by consulting a third reviewer (CF).

### Quality appraisal

The 10-item Critical Appraisal Skills Programme (CASP) checklist was used to appraise the methodological quality of included studies [23]. The CASP tool is widely used in healthcare-related qualitative evidence synthesis [24, 25] and is endorsed by the Cochrane Qualitative and Implementation Methods Group [26]. Two reviewers (ÍO’S and KR) independently appraised the quality of each study with disagreement resolved through involvement and discussion with a third reviewer (CF).

### Data extraction and synthesis

The qualitative synthesis was underpinned by the seven phases of meta-ethnography as originally developed by Noblit and Hare [20]. This is one of the most commonly utilised qualitative synthesis methods in healthcare research and is an iterative process, which focuses on the development of analytical rather than descriptive findings [27]. Phase one, ‘getting started’, involved describing the gap in research and development of the research question followed by phase 2, ‘deciding what is important’, which focused on a systematic search of the literature and quality appraisal of included studies. In phase three, ‘reading the studies’, two reviewers repeatedly read the included studies to ensure familiarisation with the key concepts or metaphors before extracting first- and second-order constructs i.e. participant quotes and/or concepts developed by authors of primary studies, respectively, into NVivo 12 Pro software. Phase four, ‘determining how the studies are related’ involved juxtaposing concepts from each study against one another using a grid format, which served as the foundation for phase five, ‘translating the studies into one another’. In phase six, ‘synthesising translations’, a line-of-argument was synthesised from third-order constructs. The line-of-argument is described as *“making the whole into something more than the parts alone imply”* [20]. In the current study, the whole refers to the experiences of CGA in an inpatient hospital setting from the perspective of multiple stakeholders. The final phase ‘expressing the synthesis’ was achieved through writing up the results for dissemination. Phases four through six were led by ÍO’S with critical feedback provided throughout by KR.

## Results

### Study identification

The literature search yielded 5,165 records. Following removal of duplicates, title and abstract screening was completed on 3,526 articles and 73 articles were selected for full-text critical reading (Additional file 3). Eleven studies recruiting 301 participants met inclusion criteria and were included in the meta-ethnography.

### Descriptive characteristics of included studies

Table 1 outlines the details of the 11 included studies. The studies dated from 2011 to 2021; four were conducted in the United Kingdom [28–31], two in the Netherlands [32, 33], two in Sweden [34, 35], and one in Australia [36], Germany [37] and France [38]. With regards to population of interest, five studies were conducted with HCPs only [31, 33, 35, 37, 38], two included HCPs, older adults and caregivers [29, 36], three included both older adults and caregivers [28, 30, 32], and one included older adults only [34]. The 11 studies in this meta-ethnography reported on the experiences of 153 HCPs, 91 older adults, and 57 caregivers. The study with the smallest number of participants was Charalambous and colleagues (*n* = 8) [31] while the study with the largest number of participants was Harvey and colleagues (*n* = 61) [36]. All studies were conducted in an inpatient hospital setting with one study specifically focusing on the subspeciality area of oncogeriatrics [38]. While there was variation in the team composition and process of CGA across studies, nine studies reported a geriatrician-led model of care [28–30, 32–37].

### Quality appraisal

A summary of the results using the 10-item CASP tool for quality appraisal are presented in Table 2. Five of the 11 studies exclusively employed qualitative methods [31, 35–38], while six studies employed mixed methods [28–30, 32–34]. Of note, three of the mixed methods studies were conducted alongside a randomised controlled trial, which evaluated the impact of CGA in an inpatient hospital setting [28, 30, 34]. All 11 studies were clear in the aim of their research and the methodology utilised were appropriate. Only four studies addressed the relationship between researcher and participant [28, 30, 32, 37] and three studies provided an insufficient description of the data analysis process [32, 33, 37]. However, given the ongoing debate regarding the application of appraisal methods in the field of qualitative research [39], no studies were excluded on the basis of quality.

### Synthesis

The analysis produced three themes (third-order constructs): (1) HCPs, older adults and caregivers report conflicting views on CGA as a holistic process, (2) most HCPs, but only some older adults and caregivers, experience CGA goalsetting and care planning as collaborative, and (3) all stakeholders value continuity of care during the transition from hospital to home but often fail to achieve it. An overview of each study’s contribution to the themes is outlined in Table 3.

### Theme 1: Conflicting views on CGA as a holistic process

While there was consensus across HCPs about CGA as a holistic process to meet older adults needs, older adults and caregivers had varied experiences of CGA as holistic and weren’t always clear about the overall aim of CGA.

HCPs described CGA as enabling them to view the older adult as a whole and unique person with specific needs, wishes, preferences and resources across studies [29, 33, 35–37]. This approach ensured the focus of assessment and intervention was “*less organ focused”* [35] with greater emphasis placed on complex health problems and the interplay with functional abilities [29, 35, 38]. HCPs also reported the importance of obtaining an understanding of the health event that precipitated admission as part of holistic consideration of the older adult:

*“I think that this is one of our characteristics too, that we take in the whole person: How it was before (hospitalization), as this is really important for how we should think ahead.”* [35].

Working as part of a multidisciplinary team (MDT) and collaborating with the older adult was described as a central tenet of CGA by HCPs that enabled a holistic assessment. Team working and collaboration with older adults was viewed as non-hierarchical [29, 33, 35, 37] and *“overrunning traditional hierarchical profession-based structures”* was described as a key feature of team interaction [35]. Team working was enhanced by coordination and completion of joint assessments and use of structured proformas [29, 35, 37], which made it possible to include various perspectives and interpretations of the information yielded by CGA:

“*I think that’s partly the advantage of using multiple pairs of eyes to assess the patient. It’s often only by taking into account different types of information that you find the right way to the patient*.” [37].

HCPs described a style of team working that built on the varied disciplinary skills and expertise [29, 33, 35, 37, 38] and, in some instances, used a more distributed form of leadership and responsibility than the traditional genesis of the CGA [29].

One contrasting HCP account was presented in a study focused on oncogeriatrics where cancer specialists reported consulting with geriatricians regarding medical decisionmaking; however, challenges such as geriatricians having a less important, ambiguous and constantly negotiated position in teams led by medical oncologists were also noted [38]. In another study, a suboptimal electronic documentation system was seen by HCPs as unreliable and inefficient and created a barrier to MDT cooperation [37].

Older adults and caregivers also experienced CGA as holistic, inclusive, and person-centred in some studies [29, 32, 36] with CGA credited as playing a critical role in the management of complex needs:

*“…they listened and they treated the whole person not just her actual thing that she went in for.”* (Caregiver, male) [36].

Accounts demonstrated ways that holistic assessments had been perceived to lead to direct interventions delivered by HCPs including management of medical problems, medication reconciliation, and restoration of functional abilities [29]. For some older adults a medically focused assessment encompassing diagnostic investigations, a physical examination and subsequent monitoring of medical stability aligned with their expectations of CGA, with reports suggesting that they valued this approach:

*“I was told I would have X-rays, I would have blood taken, I would have examinations. . to discover what problems I have. Which is what it is all about, really. . to throw up something that might provide a clue, to sort of make the situation better.”* (Older adult, male) [29].

Three studies reported contrasting experiences of older adults and caregivers [28–30]. In one study, caregivers perceived that a physical examination dominated over broader dimensions of assessment, that they would have valued:

*“I guess what I was expecting was for someone to see if. . there’s any additional help that can be offered. This was more on the medical side, just a couple of ‘obs’ [observations], yeah, blood pressure and checking the blood readings, sugar levels, that’s it.”* (Caregiver, female) [29].

In the further two studies, older adults and caregivers reported a nebulous understanding of the overall aim of CGA [28, 30] and did not recognise it as a process of assessment and planning that involved them or was holistic. Perceptions of assessment and treatment were reported to be limited to *“just monitored and observed”* [30] during their period of hospitalisation. This statement was further illuminated in the reported experience from an older adult when asked specifically about his treatment:

*“Well, nothing really. Just monitoring. Just had observations every hour or so, blood pressure, being diabetic they come and took my erm sugar level every now and again, examined me two or three times, but, never had any medication other than my tablets which I took in with me.”* (Older adult, male) [30].

Caregivers also reported prompting HCPs to complete aspects of functional assessments, for example of their relative’s mobility and activities of daily living (ADL) [29].

Furthermore, discrepancies were reported between HCPs assessments and caregiver observations with an insufficient acknowledgement of concerns by HCPs:

*“I say, ‘He’s still confused, because he said some strange things to me’, and [the nurse] says, ‘Well, we haven’t noticed that’.”* (Caregiver, male) [28].

**Table 1:** Descriptive characteristics of included studies

Citation and setting	Population	Sample	Research aim(s)	Methodology (refers to overall design in studies with multiple phases, data collection and analysis)	Description of the CGA process	Team composition
Åberg et al. 2017 [35] Sweden	Healthcare professionals working across four geriatric wards (69 beds) providing care to older adults aged ≥ 65 years.	Total sample: 32 Occupational therapist (*n* = 3) Dietitian (*n* = 1) Social worker (*n* = 3) Physician (*n *= 8) Physiotherapist (*n* = 4) Registered nurse (*n* = 7) Assistant nurse (*n* = 6)	To study the perceptions of health care professionals on factors that may facilitate or hinder the quality of hospital-based geriatric care from an interdisciplinary team perspective.	Qualitative focus group interview Thematic framework approach	Interdisciplinary CGA led by a geriatrician. Assessment and care was proactive and goal-oriented with a focus on systematic preventive care processes. Specific team members were responsible for the care of each patient, which influenced the care procedures.	Geriatricianled team inclusive of registered nurses, nurse assistants, physiotherapists, occupational therapists, social workers with access to a dietician and speech therapist, as indicated.
Busetto et al. 2017 [37] Germany	Healthcare professionals working in a 150-bed geriatric hospital providing care to older adults aged ≥ 65 years with a range of diagnoses.	Total sample: *n* = 15 Medical doctor (*n* = 4) Occupational therapist (*n* = 4) Neuropsychologist (*n* = 3) Physical therapist (*n* = 2) Registered nurse (*n* = 2)	(1)To describe the implementation of an integrated geriatric care intervention at a German geriatric hospital, and (2) to explore whether the application of an integrated geriatric care model provides insights into when and why beneficial outcomes can be achieved.	Qualitative semi-structured interviewcontext-mechanisms-outcomes (CMO) based model	Multidisciplinary CGA with a focus on standardised assessments and weekly team meetings. Assessment findings formed the basis of discussions at team meetings and further care planning.	Geriatrician-led team inclusive of occupational therapists, neuropsychologists, physical therapists, nurses, speech therapists and social workers.
Charalambous et al. 2016 [31] United Kingdom	Registered nurses working on acute medical healthcare of the older person wards in a large acute hospital trust in England.	Total sample: *n* = 8 Registered nurse (*n *= 8)	To understand how documentation may affect the quality of care in one acute hospital trust in England by understanding the experiences of registered nurses towards the documentation used while working in older person care in acute hospitals wards.	Qualitative semi-structured interview Thematic content analysis	The CGA process specific to the study site not reported.	Team composition not reported.
Darby et al. 2020 [30] United Kingdom	Older adults and their informal caregivers discharged directly home from an acute medical unit within 72 hours of attending hospital.	Total sample: *n* = 24 Older adult (*n* = 18) Caregiver (*n* = 6)	To gain an in-depth understanding of the older patient and informal carer experience of an acute medical unit stay and their experience of receiving the additional intervention from geriatricians.	Mixed methods Constructivist epistemology Semi-structured interview Thematic analysis	CGA delivered by a geriatrician who aimed to coordinate the delivery of additional immediate care or aftercare they deemed necessary. Such care could include a review of diagnoses; a drug review; further assessment at home or in a clinic or by recommending admission rather than discharge; advance care planning; or liaison with primary care, intermediate care, and specialist community services. The geriatricians liaised with hospital and community health professionals with the aim of enabling the CGA process to be delivered across the interface between the acute medical unit and the community.	Specialist geriatric medical assessment (either senior trainees or fully qualified specialists) in addition to the treatment routinely provided by the units’ consultant physicians and medical team. Patients were referred to a multidisciplinary team (physiotherapist, occupational therapist), as indicated.
de Vos et al. 2017 [33] Netherlands	Healthcare professionals working in 450-bed regional hospital with a 22-bed geriatric unit for older adults aged ≥ 70 years	Total sample: *n* = 10 Geriatric nurse (*n* = 2) Transfer nurse (*n* = 2) Team leader (*n* = 5) Internist (*n* = 1)	To identify factors impacting on the effectiveness of the implementation of a novel program, the Prevention and Reactivation Care Program (PReCaP), which targets hospital-related functional decline and geriatric care ‘as usual’ without use of an explicit program.	Mixed methods Semi-structured interview Thematic analysis	The PReCaP offers a bundle of goal-oriented interventions aimed at the physical, social and psychological domains of functional decline including identification of patient at risk within 48 h after admission, assessment of risk factors for functional decline, consultation with patient and relatives to discuss vulnerability and risk factors, biweekly multidisciplinary meeting and provision of support and treatment to informal caregiver.	Geriatrician, nursing home physician, nurses trained in geriatric care, nurse practitioners, social workers, transfer nurses, case managers, psychiatrists, psychologists, physiotherapists, occupational therapists, and dieticians, as indicated.
Gardner et al. 2019 [29] United Kingdom	(1) Older adults and their informal caregivers admitted to Older Person’s Acute Assessment Unit or a rehabilitation ward in a 48-bed community hospital, and (2) Healthcare professionals delivering CGA across varying service models (hospital inpatient and hospital-at-home).	Total sample: *n* = 31* Older adult (*n* = 12) Caregiver (*n* = 10) Healthcare professional (*n* = 9) *sample pertains to inpatient hospital cohort only	To define and describe the structure, content and delivery of the CGA as practised in hospital and hospital-at-home based settings, from the perspective of healthcare professionals who deliver it and patients and caregivers who experience this type of health care.	Mixed methods Comparative case study Semi-structured interview Framework method	Older People’s Assessment Unit: the team identify patients from an accident and emergency department and a medical assessment unit to provide a timely, comprehensive assessment and plan of care for older patients, thereby contributing to decisionmaking about the appropriate ‘place of care’ at or near the beginning of the acute health event. The acute assessment is distributed across MDT members throughout the admission, with an average length of stay in the unit of 1.5 days. Community hospital: The team complete discipline-specific assessments within shared multidisciplinary ward notes. A standardised CGA document is not in use.	Older Person’s Acute Assessment Unit: Geriatrician-led team inclusive of registered nurses, (including a mental health nurse with experience of working with older people), physiotherapists, occupational therapists and Age UK staff. Community hospital: The rehabilitation ward was under the care of a consultant geriatrician with a frailty team based in the hospital. Composition of frailty team not reported.
Harvey et al. 2017 [36] Australia	(1) Older adults and their informal caregivers admitted to a 32-bed purpose-built Older Persons Evaluation, Rehabilitation and Assessment (OPERA) unit, and (2) Healthcare professionals working across hospital and primary care settings delivering care to older adults.	Total sample: *n* = 61 Older adult (*n* = 19) Caregiver (*n* = 19) Healthcare professional (*n* = 23)	(1) To identify ways to enhance the Geriatric Evaluation and Management (GEM) model of care, and (2) to describe the care transition experiences of older adults who transfer between subacute and primary care.	Exploratory, longitudinal case study design Semi-structured and focus group interview Thematic analysis	Delivered in a dedicated ward environment the multidisciplinary CGA focused on medical, psychosocial and rehabilitative care tailored to the older adult’s specific needs and promoted coordinated discharge planning.	Geriatrician-led team inclusive of registered nurses, nurse assistants, physiotherapists, occupational therapists and social workers.
Mäkelä et al. 2020 [28] United Kingdom	Older adults aged ≥ 65 years and their informal caregivers admitted to an acute care of the elderly ward or geriatrician-led care on a medical ward or community hospital.	Total sample: *n* = 36* Older adult (*n* = 19) Caregiver (*n* = 17) *sample pertains to inpatient hospital cohort only	(1) To explore the work of patients and caregivers at the time of an acute health event, the interface with health professionals in hospital and hospital at home, and (2) how their experiences related to the principles that underpin CGA.	Mixed methods Semi-structured interview Normalisation process theory	The CGA process specific to the study sites not reported.	Team composition not reported.
Ribbink et al. 2021 [32] The Netherlands	Older adults aged ≥ 65 years and their informal care givers admitted to a 23-bed intermediate care facility in an acute geriatric community hospital.	Total sample: *n* = 18 Older adult (*n* = 13) Caregiver (*n* = 5)	To evaluate the patient experience and satisfaction with care at an acute geriatric community hospital.	Mixed methods Semi-structured interview Thematic analysis	The CGA was conducted on admission and provided an overview of all medical, functional, psychological and social problems. The CGA was discussed during multidisciplinary team meetings and used to formulate a care plan for each patient with a focus on early mobilisation and rehabilitation.	Geriatrician-led team inclusive of nurses trained in geriatric care, nurse assistants and physiotherapists.
Sifer-Rivière et al. 2011 [38] France	Healthcare professionals working in French Oncogeriatric Coordination Units providing care to cancer patients aged > 75 years	Total sample: *n* = 32* Geriatrician (*n* = 6) Surgeon (*n* = 5) Radiotherapist (*n* = 5) Oncologist (*n* = 5) Palliative care physician (*n* = 2) Emergency physician (*n* = 2) Anaesthetist (*n* = 2) Representatives from the French National Cancer Institute (*n* = 2) *Three interviewees asked not to be recorded	To identify physicians views on the role of geriatric and oncologic tools in the organisation and management of medical oncogeriatric activities.	Qualitative Semi-structured interview Grounded theory analysis	The geriatric assessment focuses on evaluating the elderly patient’s general condition (vulnerability or very fragility) and to orient the search for signs of dependency or geriatric syndromes. The assessment procedures are perceived as complementary for medical decisionmaking based on other criteria such as autonomy, physiological reserves and cognitive capacity, etc. Variable levels of participation by geriatricians at multidisciplinary consulting meetings.	Team composition not reported.
Westgård et al. 2019 [34] Sweden	Older adults aged ≥ 75 years admitted to an acute geriatric medical ward.	Total sample: *n* = 10 Older adult (*n* = 10)	To explore how frail older adults experienced receiving CGA on an acute geriatric medical ward	Mixed methods Semi-structured interview Conventional content analysis	The CGA was person-centred and delivered by a multidisciplinary team to address the frail older adults’ multiple needs related to physical health, functional ability, psychological state, cognition, and social-environmental circumstances. Findings from the CGA were used to tailor individualised treatment and rehabilitation plans for the older adult.	The team consisted of medical doctors trained in geriatric medicine, nurses, nurse assistants, occupational therapists and physical therapists with access to a social worker and nutritionist, as indicated.

### Theme 2: Most HCPs but only some older adults and caregivers experience goal-setting and care planning as collaborative

This theme focuses on HCPs, older adults, and caregivers experiences and appraisal of participation in relational dimensions of CGA such as goalsetting and care planning.

HCPs viewed goalsetting as a central component of CGA and described the importance of early involvement of older adults and their caregivers in the process, which helped direct interventions delivered by all team members [33, 35, 37].

*‘The more and the earlier we can inform the patient and inform the relatives, the calmer things get, the easier it is to work undisturbed and the more effective we can be …”* [35].

The term *“perspective-integration”* illustrates HCPs description of including older adults needs and preferences together with assessment findings in the goalsetting process [35]. One study described use of the “*Goal Attainment Scaling”* plan, which ”*assisted in formulating individual goals, developing a personalized treatment plan, monitoring both the patient‘s and informal caregiver’s progress and adjusting the interventions in a timely manner as necessary*” [33]. While HCPs aspired to involve older adults in goalsetting in one study, they described difficulties achieving, this with reports highlighting the absence of older adults when creating goals at MDT meetings [35]. HCPs also reported factors such as cognitive impairment and medical instability as barriers to actively involving older adults in goalsetting and care planning. However, cooperation between all HCPs and regular involvement of caregivers in the care process compensated for the loss of the older adult as the primary conduit of information, due for example to cognitive or language deficits [35, 37].

One study, which focused on nurses’ experiences of documentation related to the CGA process for sharing with other HCPs reported feelings of exasperation over the excessive amount of time required to complete documentation and CGA care plans, which impacted their available time to directly interact with older adults [31]. A different experience of documentation was described in another study, where documentation was seen to make a positive contribution to the process of care planning when explicitly shared between older adults, caregivers and HCPs [29].

Relational aspects of care through personal interactions with HCPs, and the role of rapport and humour, were valued by older adults [29], which shaped their experience of *“feeling respected as a person”* [34]. Reciprocity in relationships with HCPs enabled communication and participation in decisionmaking related to older adults’ needs and wishes:

*“They asked and you could tell them what you were thinking or what you needed help with they said you can have help with this and that, and we said no we want to have help with this and that. It felt like they listened to us, and then we made a decision.”* (Older adult, male) [34].

While older adults did not recognise the terminology of goalsetting as a component of CGA [29] they experienced a sense of enhanced confidence when HCPs reinforced their progress and achievements through supportive interactions:

*“Speech therapy. . I am practising, and the nurses on the ward they say, I can make myself clear to them. . sheer persistence on my part, determination.”* [29].

However, in one study, caregivers perceived the goals set by HCPs as “*too ambitious”* and did not reflect older adults baseline level of functional abilities:

*“He had a goal of walking 500 meters, whereas he could only walk less than 100 meters preadmission.*” (Caregiver, male) [32].

Older adults described contrasting levels of involvement in decisionmaking and information sharing pertaining to care planning across three studies [28, 32, 34]. The words “*an irreversible decision made by a doctor”* illustrates the sense of feeling excluded from decisionmaking in the study by Mäkelä and colleagues [28]. In another study, some older adults described feeling *“marginalised”* from participation in decisionmaking when they were given information by HCPs but not offered an opportunity to discuss concerns related to their medical status or organisational routines on the ward or when they were not engaged in communication, informed or “*given the privilege to know what was going on*” [34].

Limited opportunity to discuss concerns with HCPs regarding *“coping with needs and risks”* in the context of post discharge support was described as a source of anxiety for some caregivers [28, 29] and resulted in a perception that care planning was unrealistic [28]. Some caregivers described an under appreciation by HCPs of the *“hidden”* support they provide, which was key to enabling older adults return home following hospital discharge:

*“They were happy that [my mother] could cook for herself and clean and get herself up, which is all fine, except she can’t because she relies pretty much on me. . she assumes I’m not included in what you call ‘help’.”* (Caregiver, male) [29].

Caregivers also described concerns regarding a lack of bidirectional communication where HCPs had not approached them during their relatives inpatient admission, including instances where a discharge plan was made without their involvement [29]. However, caregivers in one study expressed reluctance to challenge HCPs if their concerns were not acknowledged:

*“You don’t like to interfere and you don’t like to be a nuisance.”* (Caregiver, female) [28].

**Table 2:** Results of CASP quality appraisal

	CASP Criterion 1	CASP Criterion 2	CASP Criterion 3	CASP Criterion 4	CASP Criterion 5	CASP Criterion 6	CASP Criterion 7	CASP Criterion 8	CASP Criterion 9	CASP Criterion 10
Citation	Clear statement of aim	Qualitative methodology appropriate	Appropriate research design	Sampling	Data collection	Research reflexivity	Ethical consideration	Appropriate data analysis	Clear statement of findings	Research value
Åberg et al. 2017 [35]	Yes	Yes	Yes	Yes	Yes	Unclear	Yes	Yes	Yes	Yes
Busetto et al. 2017 [37]	Yes	Yes	Yes	Yes	Yes	Yes	Yes	Yes	Yes	Yes
Charalambous et al. 2016 [31]	Yes	Yes	Yes	Yes	Yes	Unclear	Yes	Yes	Yes	Yes
Darby et al. 2020 [30]	Yes	Yes	Yes	Yes	Yes	Yes	Yes	Yes	Yes	Yes
de Vos et al. 2016 [33]	Yes	Yes	Yes	Yes	Yes	No	Yes	Unclear	Yes	Yes
Gardner et al. 2019 [29]	Yes	Yes	Yes	Yes	Yes	No	Yes	Yes	Yes	Yes
Harvey et al. 2017 [36]	Yes	Yes	Yes	Yes	Yes	No	Yes	Yes	Yes	Yes
Mäkelä et al. 2020 [28]	Yes	Yes	Yes	Yes	Yes	Yes	Yes	Yes	Yes	Yes
Ribbink et al. 2021 [32]	Yes	Yes	Yes	Yes	Yes	Yes	Yes	Unclear	Yes	Yes
Sifer- Rivière et al. 2011 [38]	Yes	Yes	Yes	Yes	Unclear	No	No	Unclear	Yes	Yes
Westgård et al. 2019 [34]	Yes	Yes	Yes	Unclear	Unclear	No	Yes	Yes	Yes	Yes

### Theme 3: All stakeholders value care continuity during the transition home from hospital but often fail to achieve it

This theme relates to the experiences of CGA in an inpatient hospital setting, continuity of care, and the transition to home. While there was broad consensus across HCPs about processes and pathways that positively influenced these experiences, older adults and caregivers described heterogenous experiences of CGA across the continuum of care.

HCPs described striving for optimal continuity of care and seamless transitions from hospital to home [29, 33, 35, 38]. Gardner and colleagues described referral to the “*hospital at home”* team as an expedited means of hospital discharge while ensuring continuity of care through continued observation and assessment of the older adult in their home environment:

*“. . we go on the basis of the assessment that follows CGA principles and the people that we tend to typically refer on. . are those. . who require a home-based assessment following ‘discharge to assess’ principles. . .”* [29].

Another study described the role of a *“transfer nurse”* who discussed and coordinated options for the *“post-discharge follow-up care trajectory”* with older adults and their family as soon as a discharge date was determined by the team [33]. This aimed to minimise delays in the care process and promote a positive discharge experience.

Various fora for team coordination and cooperation such as team conferences, board rounds, and ad hoc team discussions enabled the flow of information between HCPs to be *“synchronised”* with respect to older adults’ progress and discharge plans [29, 33, 35]. Prerequisites for effective and efficient team communication were accessibility of team members to one another, open communication style, and staff continuity [35]. Åberg and colleagues highlighted the importance of timely contact with caregivers and staff in municipality care to minimise potential delays in care processes and to ensure continuity in care and support [35]. A digital file transfer system in one study supported the patient transfer process from hospital staff to post-discharge care providers avoiding the need for faxes, phone calls etc. [33].

While HCPs described efforts to achieve continuity of care across the transition process in one study, they acknowledged difficulties including system pressures and *“varying quality of information exchange between hospital and community sectors”* [36]. In another study, researchers found no evidence of how HCPs mobilised or linked with community resources to optimise continuity of care or service provision following hospital discharge [37].

Older adults and caregivers also described inclusive processes, which facilitated a seamless transition from hospital to home [29, 36]. They valued information sharing and personal contact with service providers who had a remit of bridging transitions and ensuring continuity of care between hospital and home [29, 36]:

*“…they’ve been exceptional really, because today we went into my house and had a look. A nurse went with me…just to see what I had at home.”* (Older adult, female) [36].

Other tailored strategies included pre-discharge family meetings, home visits, provision of a written discharge summary, and provision of information about onward referrals to community services, which HCPs had actioned [29, 34, 36].

Conversely, the experience of planning for discharge home was described as an aspect of CGA that was frequently associated with suboptimal communication and unmet needs by older adults and caregivers, with four studies reporting gaps in information provision regarding post discharge care and follow-up [28, 30, 32, 36]. Older adults described the ambiguity around the date of discharge as disruptive in two studies [32, 36].

*“Well the decisions about going home have been very staggered…one day it’s you’re going home and the next day it’s the next day…and all of a sudden now it was Wednesday.”* (Older adult, male) [36].

Older adults and caregivers acknowledged the challenges and time constraints imposed by the inpatient hospital setting, which they recalled, was not conducive to establishing rapport with HCPs and occasionally hindered their ability to engage in discussions regarding their health needs and discharge plan [29, 34]. Furthermore, older adults described a sense of feeling disempowered by inpatient hospital processes, which challenged their ability to engage in usual routines and ADLs [29, 30]:

*"When you go into hospital they. . take the dosette box, and then they won’t give me the tablets out of it. And until the doctor prescribes them, you don’t get them. I’ve been awake at 1 o’clock at night, waiting for my tablets."* (Older adult, male) [29].

The perception of an imperative to *“empty beds”* was felt by older adults during their period of hospitalisation, which they felt shaped the actions and decisionmaking of HCPs [28, 29]:

“*They need to get you out, need the bed, and suddenly you’re gone and there’s things missing.”* (Older adult, male) [28].

In one study, caregivers recalled a personal responsibility to re-establish links with community services to mitigate against delays in follow-up due to gaps in information provision from hospital staff to community services, after hospital discharge:

*“There always seems to be that lapse when people come out, there’s no coordination at all of various areas.”* (Caregiver, male) [28].

Many caregivers in one study commented on the lack of written summaries provided at hospital discharge, where copies of discharge summaries were provided they were primarily viewed as communication between HCPs and did not address caregivers needs related to the trajectory of the older adult’s health needs [28].

Older adults perceived they had ongoing health and ADL needs following hospital discharge [28, 30, 32, 36] and they expressed concerns about *“continuing with a pattern of fluctuating symptoms and functional capabilities”* [28] despite receiving a CGA during their acute hospital admission. They described having unacknowledged concerns about their overall health problems and timing of discharge home [28, 30], which older adults and caregivers in one study, attributed to subsequent hospital readmission:

*“The doctor came round, looked at the chart, and says, ‘Well, you can go home now’. I was just amazed. I came home, I couldn’t swallow anything. .The ambulance came out and they took me back.”* (Older adult, female) [28].

**Table 3:** Contribution of included studies towards themes

Citation	Conflicting views on CGA as a holistic assessment process	Most HCPs but only some older adults and caregivers experience goalsetting and care planning as collaborative	All stakeholders value care continuity during the transition home from hospital but often fail to achieve it
Åberg et al. 2017 [35]	X	X	X
Busetto et al. 2017 [37]	X	X	X
Charalambous et al. 2016 [31]		X	
Darby et al. 2020 [30]	X		X
de Vos et al. 2016 [33]	X	X	X
Gardner et al. 2019 [29]	X	X	X
Harvey et al. 2017 [36]	X	X	X
Mäkelä et al. 2020 [28]	X	X	X
Ribbink et al. 2021 [32]	X	X	X
Sifer-Rivière et al. 2011 [38]	X	X	X
Westgård et al. 2019 [34]		X	X

### Line-of-argument synthesis

The results of the studies reflect both a reciprocal and refutational synthesis. The incongruities and contradictions outlined in third-order constructs can be explained by the contrasting experiences of CGA across the three stakeholder groups. HCPs, older adults, and caregivers ambitions and values related to CGA often align. All three groups value care continuity, collaboration and shared decision-making. Their experiences of CGA differs however. HCPs experience CGA as enhancing holistic care, enabling involvement of older adults and caregivers in relational aspects of care and improving care continuity following hospital discharge. Some older adults and caregivers do report positive experiences of CGA; however, the predominant experience is lack of clarity about what CGA is, insufficient involvement in goalsetting and care planning, care discontinuity and unmet healthcare needs following hospital discharge.

## Discussion

The current meta-ethnography has systematically reviewed and synthesised the literature on the experiences of CGA in an inpatient hospital setting from the perspective of HCPs, older adults and those important to them. We identified three key themes: (1) HCPs, older adults, and caregivers report conflicting views on CGA as a holistic assessment process, (2) most HCPs, but only some older adults and caregivers, experience CGA goalsetting and care planning as collaborative, and (3) all stakeholders value care continuity during the transition from hospital to home but often fail to achieve it.

While HCPs described a personalised and multidimensional approach to CGA underpinned by collaborative MDT working, older adults and caregivers described heterogenous experiences of CGA. A central tenet of CGA are MDTs who identify medical, psychosocial, and functional needs and develop a coordinated and integrated plan for treatment and follow-up [40, 41]. However, three of the six studies conducted with older adults and caregivers reported a predominant experience of a medically focused assessment, which they broadly equated with diagnostic investigations and monitoring of medical stability [28–30] in contrast to HCPs descriptions of a holistic assessment. The disparate descriptions of CGA as a holistic assessment process across stakeholder groups may have been influenced by environmental and time constraints imposed by the acute hospital setting [28–30, 34, 36, 37]. A growing body of primary qualitative research highlights the value of home-based CGA for community dwelling older adults [31, 42–44]. The home environment in contrast to the hospital setting is described as supporting rapport building between older adults and HCPs and studies have noted that older adults valued the additional time spent by HCPs conducting CGA in the home environment [43, 44]. Although evidence to support the effectiveness of CGA in hospital settings is compelling [8], findings from this synthesis suggest there is further work needed to ensure a holistic assessment of older adults is realised.

We found that while all stakeholders share similar ambitions and values with respect to relational aspects of CGA, this is not always reflected in older adults or caregivers’ experiences and often resulted in lack of understanding around goalsetting and coherence in care planning. Findings from our synthesis on lack of older adult and caregiver involvement in goalsetting reflects findings of the Cochrane review of 29 randomised trials on the effectiveness of CGA for older adults admitted to hospital, where less than half of the trials reported goal-setting with older adults and caregivers as a key intervention component [8]. Despite innovations in international healthcare systems seeking to expand the application of CGA [17, 18, 45], older adults and caregivers’ level of participation in the management of acute illness and their interface with HCPs is not always recognised [46, 47]. Our findings draw parallels with those outlined in a scoping review of 13 studies, which explored informal caregivers’ views on their perceived levels of involvement in collaborative discussions with HCPs [48]. Results found that HCPs do not always explicitly take into account their views, roles and responsibilities when collaborating with them. The construct of shared decision making is well established in the literature [49] and facilitates a style of healthcare communication between older adults and HCPs when decisions have to be made regarding desired care and preferred health outcomes [50]. Application of shared decision making to everyday CGA practices and processes has the potential to enhance older adults and caregivers’ satisfaction with CGA in an inpatient hospital setting.

HCPs outlined processes of care and pathways that aimed to positively influence care continuity during the transition from hospital to home; however, older adults, and caregivers descriptions largely reflected a sense of fragmented care and challenges pertaining to management of fluctuating symptoms and functional abilities following hospital discharge. The vulnerabilities and adverse outcomes older adults are exposed to during points of care transition are well established in the literature [51–53]. In keeping with the World Health Organisation’s vision for integrated care [54], CGA in an inpatient hospital setting has the potential to optimise successful transitions of care through enhanced care coordination.

### Implications for research and clinical practice

In our meta-ethnography, only two of the six studies that were conducted with older adults included older adults with cognitive impairment [28, 30]. Given that up to 40% of hospitalised older adults have some form of cognitive impairment and are twice as likely to experience adverse events during hospitalisation such as sepsis, falls, or pressure ulcers [55], future primary research studies should include this patient cohort to ensure their experiences of acute care and specific needs are sufficiently understood and met. A recent qualitative study of 18 families representing seven older adults with cognitive impairment and 20 family members, reported a diverse range of acute care experiences, which vacillated between supportive and unsupportive hospital infrastructure and resources, comprehensive and fragmented integration of care processes, as well as variable levels of person- and family-centredness [56]. These findings corroborate the need for a deeper understanding of acute care processes from the perspective of older adults with cognitive impairment and their caregivers.

Findings outlined in our meta-ethnography elucidate the divergent accounts and experiences of inpatient CGA across the three stakeholder groups, which have implications for understanding the scope of CGA as well as system-wide innovations that translate evidence into practice. These divergent findings should act as a prompt for researchers to explore engagement in structured or unstructured observation of inpatient CGA practices and processes as an adjunctive qualitative research method [57] when carrying out prospective qualitative research on the subject area. By documenting fieldwork notes on commonplace practices through observations, researchers gain greater insight into the influence of the physical environment [58], which may augment or contextualise data collected through interview methods.

CGA is considered a person-centred process and is both therapeutic and diagnostic [9]; therefore, HCPs should focus on outcomes, which older adults consider meaningful [59]. Application of the standard set of outcome measures developed and recommended by the International Consortium for Health Outcomes Measurement [60] for older adults may assist HCPs in aligning assessment and intervention practices with older adults’ needs and preferences, thereby ensuring CGA remains person-centred. Furthermore, effective communication is bidirectional between older adults and HCPs; if either the older adult or HCP is unclear about the information conveyed, the quality of the care delivered is undermined [61]. By better understanding older adults’ preferences and perspectives regarding how their clinicians communicate and share information with them, HCPs can continue to improve their communication with their patients and those important to them [62].

### Strengths and limitations

To the best of our knowledge, the current meta-ethnography represents the first systematic review and qualitative synthesis of the existing literature focused on multiple stakeholder experiences of CGA in an inpatient hospital setting. The synthesis was methodologically robust and adhered to the 19 criteria outlined in the eMERGe meta-ethnography reporting guidance [22]. Meta-ethnography, by design, is predicated on interpretative analysis rather than aggregative description of included studies; therefore, other researchers may have drawn different conclusions from the data. However, to add scientific rigor to the process of derivation of third-order constructs, two reviewers (ÍO’S and KR) engaged with the primary studies included in the synthesis over a prolonged period of time. Furthermore, a numerical approach was applied to the analytic process whereby the number of studies contributing to each third-order construct was noted. Constructs were illustrated through use of quotations, which reflected both the reciprocal and refutational translation of studies [63]. Comparable and conflicting accounts of reported experiences across stakeholder groups and studies were explored throughout, which is a key strength of meta-ethnographic synthesis [27].

However, our synthesis is not without limitations. We limited our inclusion criteria to English language studies only; therefore, additional published studies where English was not the chosen language may exist. All included studies were conducted in high income countries [64], which may limit generalisability to other international healthcare systems. Given that only 11 primary studies, reporting the experiences of 301 participants, across three stakeholder groups were included, underdeveloped theories and/or concepts may have been a feature during phase six, ‘synthesising translations’. However, there is currently no consensus on what constitutes too few or too many studies in a meta-ethnographic synthesis [65].

## Conclusion

This meta-ethnography synthesised the experiences of CGA in an inpatient hospital setting from the perspective of HCPs, older adults, and those important to them. While HCPs, older adults, and caregivers’ values and ambitions related to CGA broadly align, their experiences often differ. The identified themes highlight organisational and relational dimensions of care, which positively and negatively influence CGA practices and processes. Findings underscore the importance of involving older adults and those important to them as partners across the continuum of care.

### Supplementary Information

**Additional file 1. **eMERGe checklist

**Additional file 2. **Search string for electronic databases

**Additional file 3**. PRISMA flow

## Data Availability

The dataset used and analysed during the current study can be obtained by contacting the corresponding author, Ide O’Shaughnessy

## Abbreviations

CGA: Comprehensive Geriatric Assessment
MDT: Multidisciplinary Team
CASP: Critical Appraisal Skills Programme
ADL: Activities of Daily Living
