# Supplementary material for: Stakeholders’ experiences of comprehensive geriatric assessment in an inpatient hospital setting: a qualitative systematic review and meta-ethnography
Source: BMC Geriatr. 2023 Dec 8;23:821. doi: 10.1186/s12877-023-04505-w (PMC10704800; doi:10.1186/s12877-023-04505-w)
Supplement: Supplementary file 2 — Additional file 2. Search string for electronic databases [file 12877_2023_4505_MOESM2_ESM.docx]

| S1 | CGA OR Geriatric Assessment OR comprehensive geriatric assessment OR geriatric evaluation OR Geriatric consultation OR multidisciplinary assess* OR multi‐disciplinary assess* |
| --- | --- |
| S2 | Qualitative OR Experience* OR Perspective* OR case stud* OR interview* OR focus group* OR participant observation OR Naturalistic observation OR ethnograph* OR phenomenol* OR lived experience OR grounded-theor* OR thematic analysis OR Narrative analysis OR Discourse analy* OR field-notes |
| S3 | S1 AND S2 |
