# Supplementary material for: Stakeholders’ experiences of comprehensive geriatric assessment in an inpatient hospital setting: a qualitative systematic review and meta-ethnography
Source: BMC Geriatr. 2023 Dec 8;23:821. doi: 10.1186/s12877-023-04505-w (PMC10704800; doi:10.1186/s12877-023-04505-w)
Supplement: Supplementary file 3 — Additional file 3. PRISMA flow [file 12877_2023_4505_MOESM3_ESM.docx]

**Identification of studies via databases**

Duplicates removed

(n = 1,639)

Records identified through database searching

(n = 5,165)

**Identification**

Records screened

(n = 3,526)

**Screening**

Records excluded

(n = 3,450)

Reports assessed for eligibility

(n = 76)

Full-text article excluded

(n = 65)

| Study design  Quantitative findings (n = 16)  Population and setting  Not an in-patient hospital setting (n =29)  Surgical in-patient cohort (n = 5)  Intervention  Did not meet CGA criteria (n = 15) |
| --- |

**Eligibility**

Studies included in qualitative synthesis

(n = 11)

**Included**
